# Pathway Activity Score Learning for Dimensionality Reduction of Gene Expression Data

**DOI:** 10.1007/978-3-030-61527-7_17

**Published:** 2020-09-19

**Authors:** Ioulia Karagiannaki, Yannis Pantazis, Ekaterini Chatzaki, Ioannis Tsamardinos

**Affiliations:** 8 University of Bari Aldo Moro, Bari, Italy; 9 Aristotle University of Thessaloniki, Thessaloniki, Greece; 10 Open University of Cyprus, Nicosia, Cyprus; 11 Dalhousie University, Halifax, NS Canada; 12 Department of Computer Science, University of Crete, Heraklion, Greece; 13 Institute of Applied and Computational Mathematics, Foundation for Research and Technology - Hellas, Heraklion, Greece; 14 Laboratory of Pharmacology, Medical School, Democritus University of Thrace, Alexandroupoli, Greece; 15 Institute of Agri-food and Life Sciences, University Research Center, Hellenic Mediterranean University, 71410 Heraklion, Crete Greece; 16 Gnosis Data Analysis PC, Heraklion, Crete Greece

**Keywords:** Pathway activity, Dimensionality reduction, Disease classification, Differential activation analysis

## Abstract

Molecular gene-expression datasets consist of samples with tens of thousands of measured quantities (e.g., high dimensional data). However, there exist lower-dimensional representations that retain the useful information. We present a novel algorithm for such dimensionality reduction called Pathway Activity Score Learning (PASL). The major novelty of PASL is that the constructed features directly correspond to known molecular pathways and can be interpreted as pathway activity scores. Hence, unlike PCA and similar methods, PASL’s latent space has a relatively straight-forward biological interpretation. As a use-case, PASL is applied on two collections of breast cancer and leukemia gene expression datasets. We show that PASL does retain the predictive information for disease classification on new, unseen datasets, as well as outperforming PLIER, a recently proposed competitive method. We also show that differential activation pathway analysis provides complementary information to standard gene set enrichment analysis. The code is available at https://github.com/mensxmachina/PASL.

## Introduction

Molecular data, such as gene expressions, are often very high dimensional, measuring tens of thousands molecular quantities. For example, the Affymetrix micro-array platform GPL570 for humans measures the expressions of 54675 probe-sets, corresponding to all known human genes. As such, visually inspecting the data, understanding the multivariate gene correlations, and biologically interpreting the measurements is challenging. To address this problem, several methods have appeared that reduce the dimensionality of the data. Dimensionality reduction (a.k.a. latent representation learning) constructs new dimensions (features, quantities, variables). The purpose is to reduce the number of features making them amenable to inspection while maintaining all “useful” information. For example, consider the representation of music. The raw data (original measured quantities) correspond to the sound spectrum which is visually incomprehensible to humans. However, music at each time-point can be represented as a sum of prototypical states (notes) and musical scores, which are much more intuitive. Similarly, we can ask the questions: Are there prototypical cell states whose sum can represent any cell state (e.g., gene expression profile)? What are the “notes” of biology? How can we learn such representations automatically?

Numerous dimensionality reduction techniques have been proposed. Some of the most prevalent ones are arguably the PCA, Kernel PCA [15], t-SNE [11], and Neural Network autoencoders. All of these methods learn a lower dimensional space (latent space) of newly constructed features and represent the data as a linear combination of those. The projection aims to retain the data variance and exhibit a low data reconstruction error. However, the data representation in the new feature space is biologically unintepretable. To improve interpretability other methods introduce sparsity to the latent space in the sense that new features are constructed as linear combinations of only a few of the original molecular quantities. Such methods are the Sparse PCA [20] and sparse variants of Non-negative Matrix Factorization [10] for molecular data [4, 6]). The new constructed features are sometimes called *meta-genes* [3]. Any clustering method could also be defined as creating meta-genes and new features. However, *the meta-genes are still hard to interpret biologically as they do not directly correspond to the known biological pathways or other known gene sets*.

In this work, we develop a novel method for unsupervised feature construction and dimensionality reduction based on the availability of prior knowledge, called Pathway Activity Score Learning or **PASL**. PASL aims at a trade-off between biological interpretability, and computational performance. PASL accepts as input a collection of predefined sets of genes, hereafter called **genesets**, such as molecular pathways or gene ontology groups. It has two phases, the *inference phase* and the *discovery phase*. During the inference phase, **PASL constructs new features that are constrained to directly correspond to the available genesets.** The new features could be thought as **activity scores** of the corresponding genesets. The inference phase ends when it has captured as much information as possible (maximum explained variance) given only the provided genesets. However, a large percentage of the measured quantities is not mapped to any known genesets. In the discovery phase, PASL constructs features that are not constrained to correspond to the given genesets trying to capture the remaining information (variance) in the data.

We evaluate PASL in two sets of computational experiments. (a) We use two collections of real micro-array gene expression datasets, one for Breast Cancer and one for Leukemia. *It is shown that PASL learns latent representations that allow it to perform predictive modeling based on the novel features. The computational experiments are performed on test datasets never seen by PASL during feature construction*. Predictive modeling uses an AutoML platform for molecular data called Just Add Data Bio or **JADBIO** [17] that searches thousands of machine learning pipelines to identify the optimally predictive one and estimates the out-of-sample predictive performance of the final model in a conservative fashion. *Analysis in the new feature space is orders of magnitude faster than the one performed using the original feature space*. In addition, the resulting predictive models are on par and often outperform the ones constructed using the original molecular quantities. PASL is compared against PLIER [12], arguably the algorithm closer in spirit to PASL. PASL outperforms PLIER in terms of predictive performance.

In the second set of computational experiments, (b) we show that PASL’s constructed features can complement standard gene set enrichment analysis (**GSEA**). Specifically, the geneset activity scores output by PASL can be employed to perform differential activation analysis (**DAA**) and identify the genesets that behave differently between two different classes (e.g., cases vs controls, or treatment vs controls). Conceptually, this is equivalent to gene differential expression analysis that identifies genes whose expression behaves differently in two classes. Our experiments indicate that DAA complements GSEA: it can identify genesets that are not identified by GSEA as statistically significant. Moreover, DAA has larger statistical power than GSEA and, in general, it identifies the affected genesets with lower *p*-values than GSEA.

## Pathway Activity Score Learning Algorithm

### Preliminaries

The PASL algorithm accepts as input two 2D matrices *X* and *G*. Matrix  contains the molecular measurements, where *n* is the number of samples and *p* the number of features. Typically $n \ll p$. For micro-array gene expression data, the rows of *X* correspond to molecular profiles while the columns to the gene expressions of the probe-sets. Hereafter, *we will refer to probe-sets as genes for simplicity, unless otherwise noted; however, the reader is warned that there is not a one-to-one correspondence between probe-sets and genes.* PASL also accepts a gene membership matrix $G \in \{0, 1\}^{g\times p}$ with *g* being the number of predefined groups of genes. Each row of *G*, denoted by $\mathbf {g}_i$ for the *i*-th row, corresponds to a molecular pathway, gene ontology set, or any other predefined gene collection of interest called **geneset** hereafter. We set $G_{ij} = 1$ if gene *j* belongs to the *i*-th geneset, and 0 otherwise.

PASL assumes the data *X* can be decomposed as:  , where  is a sparse matrix. In other words, each molecular profile at row *j* of *X* is a linear combination of rows of *D* with coefficients in the *j*th row of *L* with an isotropic noise added to it. *D* is called the **dictionary** and its rows the dictionary **atoms**, denoted with $\mathbf {d}_i$. Given training data *X*, PASL outputs the two matrices *D* and *L*. *D* is the concatenation of two sub-dictionaries $D_1$ and $D_2$ ($D = [D_1 ; D_2]$) with dimensions $a_1\times p$ and $a_2\times p$, respectively (hence, $a = a_1 + a_2$). $D_1$ *is a dictionary where each atom* $\mathbf {d}_i$ *is constrained to correspond to only one geneset of the matrix* *G*, in the sense that the non-zero elements of $\mathbf {d}_i$ correspond to the genes in the particular geneset. Thus, $D_1$ is the part of the dictionary that is biological interpretable. $D_2$ is just a sparse dictionary meant to explain the remaining variance of the data and suggest the existence of yet-to-be-discovered genesets. $D_1$ is the outcome of the first phase of PASL, called **inference phase**, while $D_2$ is the outcome of the second phase, called the **discovery phase**.  is the representation of the data in the latent feature space (*PASL scores*). It provides the optimal projection of *X* on the row space of *D* and it is computed by minimizing the Frobenius norm between *X* and $L\cdot D$.

### Inference Phase

One approach to extract the genesets with the highest variance in the dataset is to restrict the data matrix to the features that correspond to a pathway, estimate the first principal component, repeat the same for all pathways and then keep the principal component with the highest variance *(dynamic approach)*. We mathematically formulate this problem as1where $X(:,\mathbf {g}_i)$ denotes the data matrix restricted by the *i*-th geneset. Then, we add the $i^*$ principal component to the dictionary, remove its contribution from the dataset and repeat the same procedure until a pre-specified criterion is met. The described algorithm is guaranteed to return an ordered dictionary whose atoms have the highest variance. Nevertheless, it can be prohibitively expensive in terms of computational cost since at each iteration it computes thousands of principal components that are discarded. In order to remedy the computational burden, one solution could be to pre-compute the principal components for all restricted-to-the-pathways data matrices, then, order them and keep the principal components with the highest variance (*static approach*). Despite being relatively computationally efficient, this approach does not necessarily lead to an optimal solution. Specifically, the ordering of the genesets is fixed, but at each iteration the data matrix changes because the contribution of each new atom is removed from it. This might affect the actual ordering of the variance, hence the optimality of the solution.

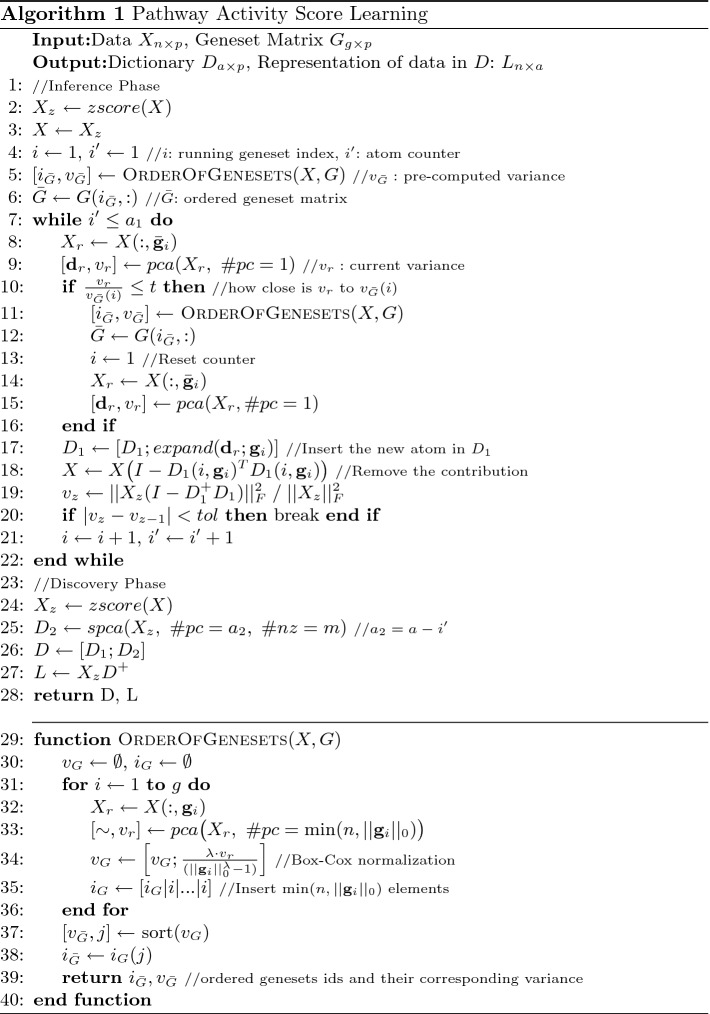



The inference phase of PASL shown in Algorithm 1 (lines 1–22 and 29–40) balances between the dynamic and the static approach. As in the static approach, it computes the ordering of the principal components’ variance (lines 5 and 29–40) and iteratively select the atoms based on this ordering (while loop; lines 7–22). The difference between PASL and the static approach is that PASL checks how close is the current variance from the expected pre-computed variance (line 10). If the relative change is below a threshold then PASL recomputes the ordering of the principal components’ variance (lines 11–15). The hyper-parameter *t*, which takes values between [0, 1], controls how often the variance reordering is performed henceforth the proximity to optimality. The higher the value of *t* the more often the evaluation of the ordering is happening thus more accurate the dictionary in terms of explained variance is on the cost of being computationally more expensive.The stopping criterion asserts that the inference phase of PASL stops when there is no further decrease in the relative reconstruction error (i.e., the variance of the normalized residual error) (line 20). Finally, we remark that the variance values are normalized before they are ordered (line 34). This is absolutely necessary due to the wide variation of the number of genes in each geneset which varies from few dozens to few thousands of genes. We choose as normalization method the Box-Cox transformation on the number of genes and optimize over its hyper-parameter $\lambda$.

### Discovery Phase

After the inference phase where we extracted as much as possible variance from prior knowledge, we will distill the remaining variance of the data without restrictions on the location of the non-zero elements of the dictionary atoms using a sparse –hence, interpretable– dimensionality reduction technique aiming to reveal new potential pathways which were previously unknown. Based on its generality, efficiency and speed, we employ in our experiments Sparse Principal Component Analysis (SPCA) [20] (line 25 in Algorithm 1). We note though that any sparse dimensionality reduction technique can be utilized. SPCA applies both $l_1$ and $l_2$ penalties in order to regularize and enforce sparsity.

However, we do not tune the respective hyper-parameters, instead, we require the SPCA algorithm to return a fixed number of non-zero elements per atom. We denote this number with *m* and we set it to 2000 in our experiments.

### Selection of the Hyper-Parameters’ Value

**Fig. 1.**
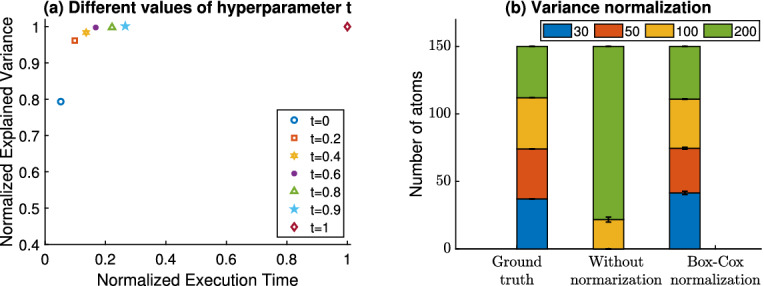
**(a)** The explained variance (y-axis) as a function of the execution time (x-axis) is shown for different values of *t*. For $0.4 \le t \le 0.9$, the execution time is reduced by a percentage between 65% and 85% with minimal impact on the explained variance. **(b)** The simulated dictionary (ground truth; left bar) consists of equally distributed pathways with 30, 50, 100, 200 genes. The middle bar shows the distribution of selected pathways when PASL is applied without normalization while the right bar shows the selected pathways when Box-Cox normalization is applied with $\lambda =1/3$. Apparently, the normalization of the variance is necessary for PASL in order to avoid being biased towards selecting genesets with a larger number of genes.

**Effect of** *t* **on the explained variance and the execution time.** The most time-consuming part of PASL is the execution of the function OrderOfGenesets in Algorithm 1 due to the large number of PCA calculations (one for each geneset). Hyper-parameter *t* controls how often the function OrderOfGenesets will be called. When $t=1$ then it is called at every iteration while it is called once at the beginning and never again when $t=0$. In order to determine the optimal value for *t*, we perform an experiment with a merged collection of microarray datasets where the total number of samples is $n=4235$, the number of genes $p=54675$ and a fixed number of atoms $a_1=200$. Figure 1(a) demonstrates the explained variance as a function of the execution time for different values of *t*. Based on this plot, we set *t* to be equal to 0.9 (cyan star symbol in Fig. 1(a)).

**Box-Cox Normalization of the Variance.** The number of genes, i.e., the number of non-zero elements in each row of the geneset matrix *G*, varies from few dozens to several thousands making the geneset ordering based on variance susceptible to such variations. Indeed, we experimentally observe that genesets with more genes tend to be selected frequently while genesets with a low number of genes were rarely selected (see also the middle plot of Fig. 1(b)). Therefore, it is essential to normalize the variance of each geneset relative to the number of genes it contains. We propose to normalize the variance using the Box-Cox transformation [2] on the number of genes (i.e., on $\Vert \mathbf {g}_i\Vert _0$) which is given by

2 $$\begin{aligned} \small y' = {\left\{ \begin{array}{ll} (y^\lambda - 1)/\lambda &{} \text { if } \lambda \ne 0 \\ \log (y) &{} \text { if } \lambda =0 \end{array}\right. } \end{aligned}$$

where $\lambda$ is a tunable hyper-parameter which controls the power scaling on *y*.

The value of $\lambda$ is determined by a targeted experiment using simulated data which are generated using genesets with both small and large numbers of genes. Simulated data are generated by first creating the prior information matrix *G* consisting of equally distributed genesets with specific number of genes. Then, we construct a dictionary using randomly selected genesets which are also equally distributed. Specifically, we create $n=400$ samples with $p=500$ features while the numbers of genes per geneset take the values 30, 50, 100, 200.

After extensive tests with different values of Box-Cox transformation hyper-parameter, we set $\lambda = 1/3$. The geneset selection results obtained with PASL are presented in Fig. 1(b). Evidently, the use of Box-Cox transformation with $\lambda =1/3$ (right bar) produced results similar to the ground truth (left bar) while PASL without normalization failed to correctly infer the true dictionary (middle bar).

## PASL Evaluation on Real Gene Expression Data

**Fig. 2.**
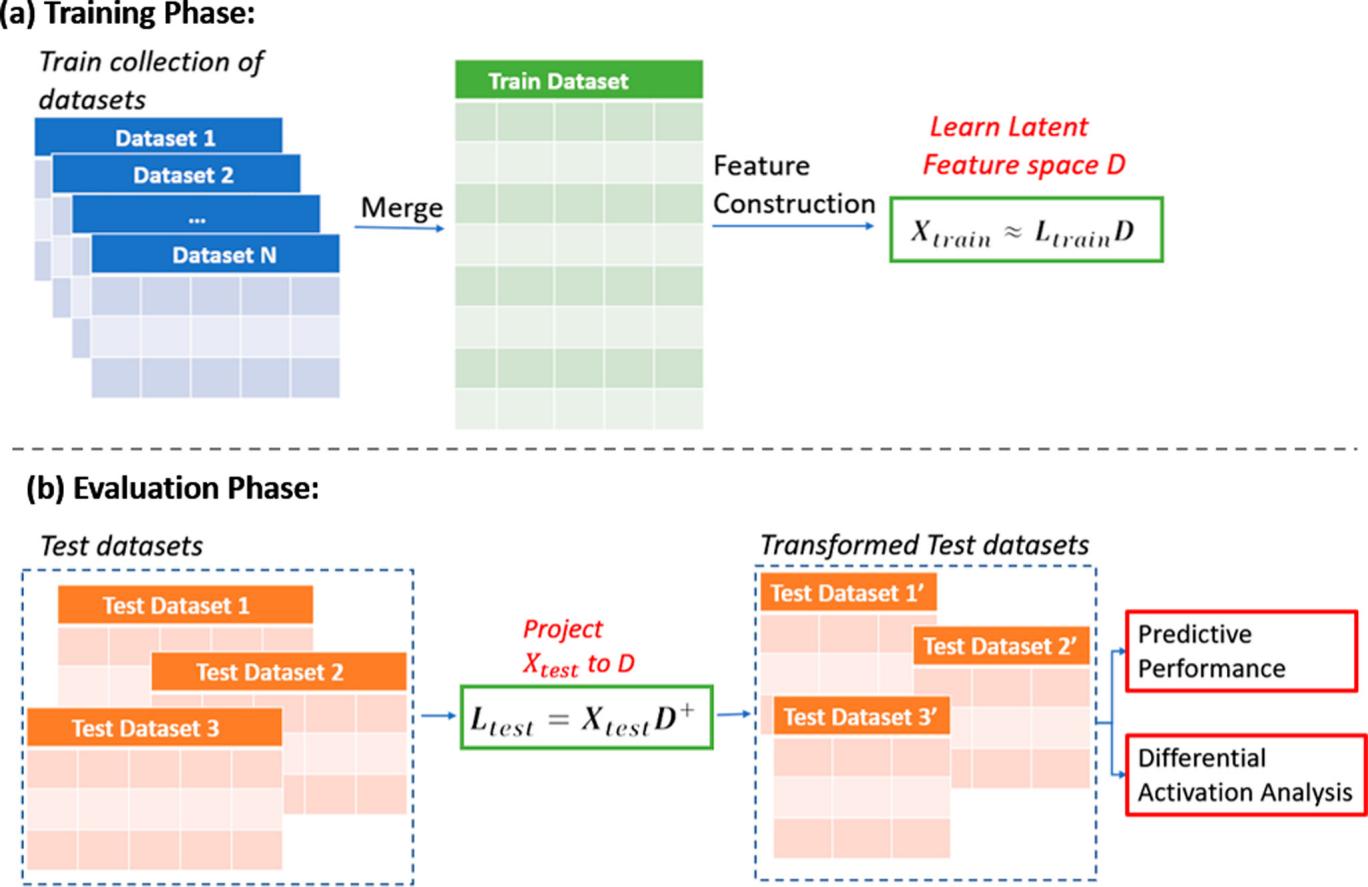
Experimental Setup. For the construction of the latent feature space, the methods are trained on a collection of gene expression datasets. The evaluation is performed on new unseen test datasets, where the recostruction ability, predictive performance and the significance of the pathways of the latent feature space are examined.

**Dataset Collections.** For our experiments we downloaded microarray datasets available in the Biodataome database [9]. Specifically, we downloaded all the available Breast cancer and Leukemia datasets as of May 2020 measured with the Affymetrix Human Genome U133 Plus 2.0 - GPL570 platform, each having at least 20 samples. The datasets form the *Breast Cancer collection* and *Leukemia collection*. For each collection we select 80% of the datasets to pool together and use them as training data. PASL and PLIER dimensionality reduction algorithms are applied on this training set to learn a dictionary matrix *D* of atoms (Fig. 2(a)). The remaining 20% of the available datasets are employed as test dataset and are *not seen by neither PASL or PLIER during training*. The selection of datasets used for the train or the test set is random, with the restriction that test datasets have to be accompanied by a discrete outcome (phenotype) for each sample, e.g., disease or mutation status or multiple phenotypes related to the diseases (e.g. rapid/slow early responder). The outcome is either binary or multiclass. The training set for the Breast cancer and the Leukemia collection contains 4200 and 5600 unique gene-expression profiles respectively.

**Provided Genesets.** In all experiments with real data, the gene membership matrix *G* includes 1974 pathways found in KEGG [7], Reactome [5] and Biocarta [14] which were downloaded from Molecular Signatures Database (MSigDB) of the Broad Institute [16].

**Constructing a Latent Feature Space with PASL and PLIER.** Applied to a training dataset $X_{train}$, PASL learns a transformation to a new feature space given data $X_{train}$ and a geneset matrix *G*. Subsequently, PASL learns a dictionary *D* and scores $L_{train}$ such that $X'_{train} \approx L_{train} \cdot D$. Each atom (row) in *D* corresponds to only one geneset in *G* or a newly discovered geneset (Fig. 2(a)). To apply the transformation to new test data $X_{test}$ one projects them to the row space of *D* by computing $L_{test} = X_{test}\cdot D^+$ (Fig. 2(b)). An important detail is that both train and test data are first standardized using the means and standard deviations of the training data; thus, the transformation does not require to estimate any quantity from the test data. This is important to avoid information when evaluating predictive performance on the transformed data.

We comparatively evaluate PASL against a recently introduced algorithm called PLIER [12]. Like PASL, PLIER learns a latent feature space that corresponds to known genesets. PLIER also accepts as input data *X* and a geneset matrix *G*. Similarly to PASL, it returns the scores *L* and the dictionary *D*, such that $X \approx L \cdot D$. PLIER accepts several hyper-parameters. The maxpath hyper-parameter indicates how many genesets an atom of *D* is supposed to correspond to. We set maxpath = 1 requesting that each atom in *D* corresponds to one and only geneset, so that the output is comparable to PASL. Unfortunately, *PLIER treats maxpath as indicative; atoms in D may correspond to the union of several genesets, even when maxpath = 1*. In that sense, the atoms in *D* are not as easy to interpret as the ones returned by PASL. PLIER also ignores genesets with fewer features than minGenes. We set minGenes = 1 so that no genesets are ignored. Finally, we note that in PLIER the scores *L* are computed as $X\cdot D^T \cdot (DD^T+\lambda _2 I)^{-1}$, where $\lambda _2$ is a parameter learned by the algorithm.

The atoms of PLIER are not as sparse as the ones output by PASL. For example, for the Breast Cancer collection analysis, the mean number of non-zero coefficients in each atom of PLIER is 25833 (almost half of the original feature size), while for PASL it is 1329. For the same number of atoms, PLIER uses more degrees of freedom (non-zero coefficients) to find a suitable transformation to a latent space. For a fair comparison in the subsequent experiments, we impose the restriction that the learned dictionaries $D_{\text{ P }LIER}$ and $D_{\text{ P }ASL}$ have approximately the same number of non-zero elements. To this end, we first run PLIER allowing it to construct a large number of atoms and estimate the number of atoms *a* required to reach approximately the same number of non-zeros as PASL. Then, we re-run PLIER constrained to produce only *a* atoms. Specifically, when PASL is restricted to 500 atoms, its dictionary contains 664695 and 700020 non-zeros for the Breast Cancer and the Leukemia collections, respectively. PLIER is limited to 29 and 30 atoms instead, producing dictionaries with 699976 and 782114 non-zeros, respectively.

### Predictive Performance in Latent Feature Space

**Fig. 3.**
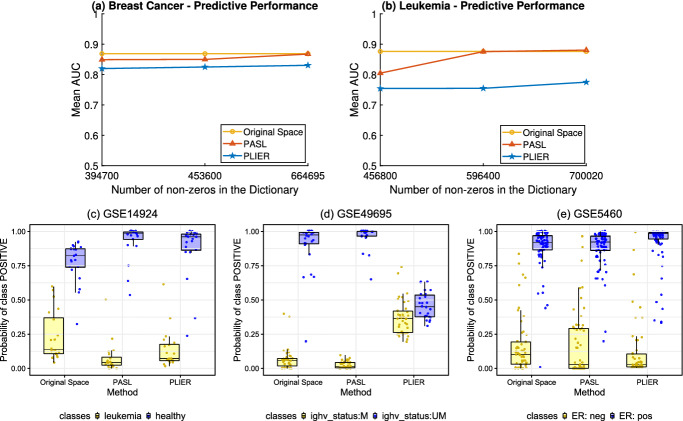
**(a), (b)** Mean AUC of Breast Cancer and Leukemia test datasets **Lower row:** Out-of-sample probability of selected results of **(c)** The best visualization for PASL vs Original, **(d)** The best visualization for PASL vs PLIER (The outcome stands for the mutation status of immunoglobulin heavy chain (IGHV) gene) and **(e)** The best visualization for PLIER vs PASL.

This set of experiments examines the following research question: *does the transformation to the latent feature space capture all important information*, defined as the information required to classify to typical outcomes (phenotypes) such as the disease state. To this end, we employ predictive modeling on the **test datasets** and estimate the predictive performance of the best identified model. Each test dataset’s outcome leads to binary or multiclass classification tasks. For the classification, we employ an automated machine learning architecture (AutoML), called **JADBIO** (Just Add Data Bio, www.jadbio.com), version 1.1.21. JADBIO has been developed specifically for small-sample, high-dimensional data, such as multi-omics data. The use of JADBIO is meant to ensure that (a) out-of-sample AUC estimates are accurate, and (b) performance does not depend on a single classifier tried with just the default hyper-parameters. Instead, for classification, JADBIO uses the SES feature selection algorithm [8], combined with ridge logistic regression, decision trees, random forests, and SVMs for modelling. It automatically tunes the hyper-parameters of the algorithms, trying thousands of combinations of algorithms and hyper-parameters. It estimates the performance of the final winning model produced by the best configuration (pipeline of algorithms and hyper-parameter values) using the BBC-CV protocol [19]. The latter is a version of cross-validation that adjusts the estimate of performance of the winning configuration for multiple tries to provide conservative AUC estimates. A detailed description of the platform along with a massive evaluation on hundreds of omics datasets is included in [17]. JADBIO has produced novel scientific results in nanomaterial prediction [18], suicide prediction [1] and others.

**Table 1.** AUC of the test datasets for PASL, PLIER and Original space (initial test datasets). PASL and PLIER are tested for approximately equal number of non-zero entries in the dictionary matrix. For Breast cancer data PASL’s latent space consists of 500 dimensions-664695 non-zeros. PLIER’s latent space consists of 29 dimensions of 699976 non-zeros. For Leukemia, PASL’s latent space consists of 500 dimensions of 700020 non-zeros. PLIER’s latent space consists of 30 dimensions of 782114 non-zeros.

Breast Cancer				Leukemia			
Data ID	PASL	PLIER	Original	Data ID	PASL	PLIER	Original
54002	0.999	1	0.995	15434	0.985	0.747	0.987
5460	0.952	0.958	0.96	14924	0.996	0.987	0.91
36771	0.935	0.933	0.963	23025	0.762	0.766	0.741
66161	0.664	0.486	0.579	21029	0.95	0.694	0.966
76124	0.976	0.98	0.97	28654	0.767	0.616	0.762
66159	0.759	0.506	0.776	14671	0.59	0.674	0.625
66305	0.513	0.569	0.535	7440	0.73	0.52	0.736
10780	0.976	0.995	0.962	66006	0.926	0.792	0.952
27562	0.835	0.776	0.914	28460	0.719	0.542	0.697
27830	0.725	0.671	0.759	26713	0.998	0.997	0.952
36769	0.953	0.963	0.96	31048	0.984	0.981	0.99
29431	0.997	0.982	0.991	39411	0.997	0.956	0.985
42568	0.991	0.975	0.927	49695	1	0.612	0.998
				50006	0.979	0.994	0.983
				61804	0.823	0.744	0.869
**Mean**	**0.8673**	** 0.830**	**0.868**	**Mean**	**0.8804**	**0.7748**	**0.876**
**Median**	**0.952**	**0.958**	**0.96**	**Median**	**0.95**	**0.747**	**0.952**

We performed classification analysis using JADBIO on 13 and 15 test datasets for Breast Cancer and Leukemia, respectively. The analysis uses the original feature space, as well as the PLIER and PASL feature spaces, for different dimensionalities. For PASL, the number of atoms to learn take the values 250, 400, and 500. The number of atoms with approximately the same number of non-zeros in the dictionary of PLIER is 20, 25, and 30. Thus, there are 7 analyses for each dataset, and $91+105$ analyses in total.*For the Breast Cancer (Leukemia) datasets 860002 (983425) classification models were trained in total by JADBIO with different combinations of algorithms and hyper-parameter values on different subsets of the input data (cross-validation).*

**Fig. 4.**
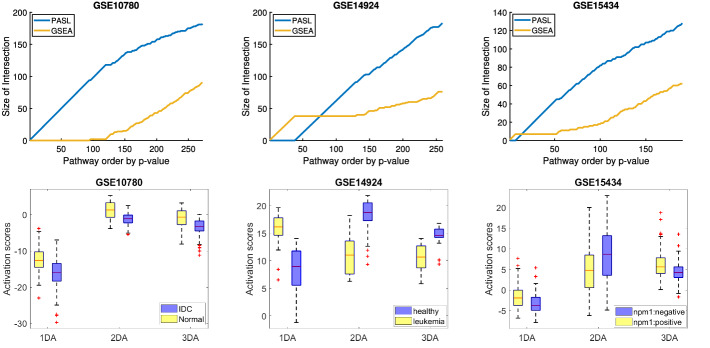
**Upper row:** Interaction plots of DAA and GSEA. The x-axis represents the total number of significant genesets. The y-axis represents the number of significant genesets that come from DAA and GSEA. **Lower row:** Box-plots of the activation scores that correspond to the first, second, third differentially activated PASL feature/pathway. It is verified that the differentially activated pathways behave differently between the phenotypes. The outcome of GSE10780 stands for Invasive Ductal Carcinoma/Unremarkable breast ducts, and the outcome of GSE15434 stands for the mutation status of Nucleophosmin 1 (NPM1).

Regarding the execution time, the analysis in the space of PASL or PLIER takes about **1 order of magnitude less time** than in the original space. The exact execution time in JADBIO depends on several factors, such as the load of the Amazon servers on which the platform runs, and thus exact timing results are meaningless. Indicatively, we mention a typical case: the analysis of GSE61804 for the original space took 1.15 h, 9 min and 5 min for PASL and PLIER respectively. Figure 3(a),(b) shows the average AUC over all test datasets for each disease for increasing number of non-zeros. **PASL outperforms PLIER and it is on par with analyses on the original space**. Thus, the learned dictionary by PASL generalizes to new test data and captures the important information to perform classification with various disease-related outcomes. At the same time, *PASL achieves 2-orders of magnitude dimensionality reduction by a sparse matrix whose atoms directly correspond to known genesets (pathways)*.

We now focus on the experiments for the largest dimension of PASL and PLIER. The number of atoms in PASL is set to 500 (664695 non-zeros for Breast Cancer, 700020 non-zeros for Leukemia). PLIER’s latent space consists of 29 (699976 non zeros) and 30 (782114 non-zeros) atoms for Breast Cancer and Leukemia respectively. Table 1 contains the detailed results for each dataset and method. The worst case (best case) for PASL is dataset with ID 27562 (14924) where it achieves 8 AUC points (8 AUC points) lower (higher) performance vs no dimensionality reduction. In contrast, there are several datasets (IDs 66161, 66159, 27562, 15434, 21029, 7440, 66006, 28460, 28460, 49695, 61804) where PLIER’s performance is lower than 10 or more AUC points.

In the lower row of Fig. 3 we visually demonstrate the ability of PASL to lead to highly predictive models. Each panel corresponds to a different test dataset. Specifically, we chose to present the visualizations from datasets that lead to the “best” visual differences for PASL vs the original space, PASL vs PLIER, and PLIER vs PASL, in Fig. 3(c)–(e), respectively. Each panel shows the box-plots of the *out-of-sample probability* of each molecular profile to belong to the positive class for the models produced in the original, PASL, and PLIER feature space. The out-of-sample predictions are calculated by JADBIO during the cross-validation of the winning model and thus, they do not correspond to the fitting of the samples used for training. The larger the separation of the distribution of the predicted probabilities, the larger the AUC.

### From Gene Set Enrichment Analysis to Differential Activation Analysis

The biological interpretability of PASL’s feature space is demonstrated in the following experiments. Since the constructed features correspond to the genesets (atoms of *D*), we can use their values (stored in the columns of *L*) to find which genesets behave differently under two conditions, e.g., disease vs. healthy or treatment vs. control. In other words, we can perform **Differential Activation Analysis (DAA)** in a similar fashion that differential expression analysis identifies the genes that behave differently. A current standard alternative method that provides insight into the underlying biology is to use Gene Set Enrichment Analysis (**GSEA**). GSEA first summarizes the probesets that correspond to the same gene e.g. by taking the minimum, maximum or average expression value. Inherently, GSEA loses information by applying this summarization and by not taking into account the covariances of the gene expressions. Subsequently, the null hypothesis is that the *p*-values of the genes in a pathway have the same distribution as the *p*-values of the genes that do not belong to the pathway.

We next examine the ability of PASL to identify genesets (pathways) that behave differently between two classes and compare it against GSEA. We employ the GSEA v4.0.3 tool from https://www.gsea-msigdb.org/gsea/index.jsp [13, 16]. We run GSEA on the test datasets in the original feature space using 10000 phenotype permutations for the permutation-based statistical test employed in the package. The input genesets are the same as the ones provided to PASL in the geneset matrix *G*. We also perform DAA on the test datasets projected to the latent space of PASL (activity scores) using the Matlab’s t-test function *mattest* with 10000 permutations. The list of *p*-values from DAA and GSEA can then be used to identify the affected pathways.

Figure 4 (upper row) shows the number of pathways identified by each method (y-axis) in the top *k* (lowest *p*-value) pathways, for each *k* (x-axis). Each panel corresponds to a different test dataset. We observe that the pathways identified by PASL have lower *p*-values and are encountered first on the list; PASL has higher statistical power in identifying some genesets that behave differently. PASL’s features correspond to pathways. The statistically significant ones are referred as *differentially activated*. Figure 4 (bottom row) visualizes why the PASL features are identified as *differentially activated*. Each panel shows the box-plots for the activation scores corresponding to the first, second, and third most statistically significant PASL feature/pathway (denoted with names 1DA, 2DA, and 3DA, respectively).

Specifically, the top 3 differentially activated pathways of GSE10780 are the “Reactome signaling by GPCR”, “Reactome Fructoce Catabolism” and “Reactome Hemostasis”. The top 3 differentially activated pathways of GSE14924 is the “Reactome metabolism of Lipids”, “Reactome Chromatin Organization” and “Reactome Gene Expression Transcription”. The top 3 differentially activated pathways of GSE15434 are the “Reactome Transport of Small Molecules”, “Reactome Developmental Biology”, “Reactome Post Translational Protein Modification”. *It is visually verified that the scores are different between the phenotypes in an easy to understand and intuitive plot.*

While DAA using PASL seems to offer several advantages (lower *p*-values, intuitive visualization), it also has a major limitation. PASL requires a training set that is related to the application (test) set. It learns atoms that only pertain to capturing information regarding the train data. For example, DAA using PASL cannot be applied to a schizophrenia dataset, before we construct a sufficiently large training dataset for the disease. As such, we consider DAA and GSEA complementary and synergistic.

## Conclusions

Molecular omics and multi-omics data are notoriously high-dimensional. Statistical or machine learning analysis of such data could hit computational obstacles due to the high dimensionality; results may be hard to interpret (e.g. interpreting thousands of differentially expressed genes or pair-wise correlations and covariances). As a result, several dimensionality reduction methods for such data have been proposed, but usually end up with an unintepretable new feature space. To the extent of our knowledge, PASL is the first technique where the new features directly correspond to prior knowledge about genesets. PASL is relatively computationally efficient by relying on a greedy, yet effective heuristic to construct the next atom. PASL projects the data to a new feature space that maintains the predictive information for a wide range of outcomes, e.g., disease or mutation status, dietary restrictions and others. The classification models created on this space outperform the ones created on the PLIER space and are on par with the ones using the original features. Classification analysis is one order of magnitude faster in PASL space than in the original space. PASL’s learned features can be used for Differential Activation Analysis identifying the pathways that behave differently between the phenotypes. This analysis is synergistic to gene set enrichment analysis, it is intuitively visualized, and often produces smaller p-values. Based on these promising results, in a future work PASL will be applied on a much larger corpus of gene expression data, spanning a wide plethora of diseases and conditions.
